# Elevated rates of HIV infection among young Aboriginal injection drug users in a Canadian setting

**DOI:** 10.1186/1477-7517-3-9

**Published:** 2006-03-08

**Authors:** Cari L Miller, Steffanie A Strathdee, Patricia M Spittal, Thomas Kerr, Kathy Li, Martin T Schechter, Evan Wood

**Affiliations:** 1 British Columbia Centre for Excellence in HIV/AIDS, Vancouver, Canada; 2 University of California School of Medicine, Department of Family and Preventive Medicine, Division of International Health & Cross-Cultural Medicine, San Diego, USA; 3 University of British Columbia, Department of Health Care and Epidemiology, Vancouver, Canada; 4 University of British Columbia, Faculty of Medicine, Vancouver, Canada

## Abstract

**Objectives:**

Recent reports have suggested that Aboriginal and American Indian people are at elevated risk of HIV infection. We undertook the present study to compare socio-demographic and risk variables between Aboriginal and non-Aboriginal young (aged 13 – 24 years) injection drug users (IDUs) and characterize the burden of HIV infection among young Aboriginal IDUs.

**Methods:**

We compared socio-demographic and risk variables between Aboriginal and non-Aboriginal young IDUs. Data were collected through the Vancouver Injection Drug Users Study (VIDUS). Semi-annually, participants have completed an interviewer-administered questionnaire and have undergone serologic testing for HIV and Hepatitis C (HCV).

**Results:**

To date over 1500 Vancouver IDU have been enrolled and followed, among whom 291 were aged 24 years and younger. Of the 291 young injectors, 80 (27%) were Aboriginal. In comparison to non-Aboriginal youth, Aboriginal youth were more likely to test seropositive for either HIV (20% vs 7%, p=< 0.001) or Hepatitis C virus (HCV) (66% vs 38%, p =< 0.001), be involved in sex work and live in the city's IDU epi-centre at baseline. After 48 months of follow-up, Aboriginal youth experienced significantly higher HIV seroconversion rates than non-Aboriginal youth, 27.8 per ppy (95% CI: 13.4–42.2) vs. 7.0 per ppy (95% CI: 2.3–11.8) respectively (log-rank p = 0.005) and the incidence density over the entire follow-up period was 12.6 per 100 pyrs (CI: 6.49–21.96) and 3.9 per 100 pyrs (CI: 1.8–7.3) respectively.

**Interpretation:**

These findings demonstrate that culturally relevant, evidence based prevention programs are urgently required to prevent HIV infection among Aboriginal youth.

## Introduction

In Canada and the United States, the respective Centres for Disease Control have been alerted to the fact that persons of Aboriginal and American Indian descent may be at elevated risk for HIV/AIDS [1-3], though little data is presently available to inform prevention efforts. In the province of British Columbia, HIV surveillance data indicated that Aboriginal people accounted for approximately 4% of the total population but comprised 18% of new HIV infections between 1996 and 2000 [4,5]. It is estimated that nearly half of the urban Aboriginal population in Canada is under the age of 25 years compared to 30% of the non-Aboriginal population[6]. To date there has been little focus on the impact of the HIV/AIDS epidemic on young Aboriginal peoples and few data are available regarding risk factors for HIV transmission among Aboriginal youth.

Aboriginal people in Canada are overrepresented among marginalized groups at risk for HIV/AIDS such as injection drug users (IDU) and street youth, particularly in the western provinces where a relatively higher proportion of Aboriginal people reside [2,6]. For example, in the Vancouver Injection Drug Users Study (VIDUS), Aboriginal people account for approximately 25% of the 1500 IDU enrolled[7]. Aboriginal service providers have suggested that injection drug use may be one of the ways in which Aboriginal people cope with the complex effects of discrimination, poverty and cultural dislocation, including the multigenerational effects of the residential school system [8].

National Canadian epidemiologic surveillance data suggest that Aboriginal youth may be at particularly high risk of HIV/AIDS, whereby 33% of newly diagnosed Aboriginal people were under the age of 30 as compared with 20% of non-Aboriginal people[2]. In addition, between 1998 and 2000, 60% of new HIV infections among Aboriginal people were attributed to injection drug use[1]. While there has been much literature documenting the explosive HIV outbreak that occurred among injection drug users in Vancouver[9], only recently has attention been paid to the elevated rates of HIV infection among Aboriginal IDUs[8,10]. However, no studies have specifically considered HIV prevalence and incidence rates among Aboriginal youth in this setting. We undertook this study to compare young Aboriginal IDU and non-Aboriginal IDU in a city where an explosive and ongoing HIV epidemic has occurred and where approximately one quarter of the IDU population are Aboriginal.

## Methods

The Vancouver Injection Drug User Study (VIDUS) is a prospective cohort study of injection drug users who have been recruited through self-referral and street outreach from Vancouver's Downtown Eastside since May 1996. The Downtown Eastside is Vancouver's poorest neighborhood and IDU epi-centre, where an estimated 4,700 IDUs and 1,000 street youth reside in an area of approximately ten city blocks, and where inexpensive housing in the form of hotels and single room occupancy hotels (SROs) are common. The cohort has been described in detail previously[9,11,12]. Briefly, persons were eligible for the VIDUS if they had injected illicit drugs at least once in the previous month, and resided in the greater Vancouver region. At baseline and semi-annually, subjects have provided venous blood samples and completed an interviewer-administered questionnaire. All participants provided informed consent, and were given a stipend ($20 CDN) at each study visit.

### Instrument

The questionnaire elicits demographic data as well as information about drug use, HIV risk behavior, and the use of drug treatment. Behavioral variables were elicited at each semi-annual follow-up visit and are in reference to the six-month period prior to the interview. Risk factor variables considered in the present analyses include: sex work, use of methadone maintenance therapy (MMT), frequency of cocaine and heroin injection, and sexual risk variables. Sex-work involvement was defined as exchanging sex for money, goods, drugs, shelter, or anything else during the previous 6 months. Sexual behaviours with casual and regular sex partners were assessed separately, and sexual risk was defined as one or more instances of unprotected vaginal or anal intercourse. Regular partners were defined as "someone you have had a sexual relationship with for more than three months (not including clients/tricks)" and casual partners were defined as "someone you have had a sexual relationship with for less than three months (not including tricks/clients)". As has been done previously[13], unstable housing was defined as living in a single room occupancy hotel, transitional living arrangements, or homelessness. As previously[11], frequent cocaine or heroin injection refers to at least daily use, and frequent crack use refers to at least daily crack cocaine smoking and we also evaluated alcohol use and reporting requiring help with injections [14] in the six months previous to the time of interview. Sexually transmitted infections (STIs) were based on self-report. All time-updated behavioral variables are elicited in reference to the six months preceding the interview whereas socio-demographic covariates (ie gender, ethnicity) were treated as fixed baseline covariates.

### Statistical analysis

The present analyses were restricted to VIDUS participants aged 24 years and younger who were recruited between May 1996 and May 2003. As has been done previously[15], youth were defined as those participants in the cohort who were ≤24 years at enrolment based on the age criterion for youth and/or adolescents used in reports on HIV/AIDS generated by the United Nations, the World Health Organization, the Centres for Disease Control and Health Canada. Aboriginal ethnicity was based on self-report to the question: "are you of First Nation, Aboriginal, Inuit, or Métis origin and/or do you have a status Indian card issued by the federal government?" For the purpose of these analyses, all Aboriginal groups were combined and defined as "Aboriginal" due to the large number of different Nations comprising Aboriginal groups in Canada and British Columbia thus presenting statistical challenges due to power related issues. However, it is noted that we present data combining individuals representing many nations that differ characteristically, and these differences are not considered in these analyses.

For the analysis of baseline characteristics and baseline HIV prevalence, we used contingency table analysis to compare socio-demographic, HIV serology, and risk factor variables for Aboriginal and non-Aboriginal youth. Chi-square and Fischer's exact tests were used to compare socio-demographic and risk factors among the Aboriginal and non-Aboriginal youth. We identified characteristics that were independently associated with Aboriginal youth by fitting a logistic regression model considering all variables that were statistically associated with Aboriginal ethnicity at the *p *< 0.05 level in univariate analyses.

Baseline HIV-negative youth with at least one follow-up visit were eligible for an analysis of the time to HIV infection. In these analyses, time zero was defined as the date of enrolment into the study. Participants who did not become HIV-infected during the follow-up period were censored as of May 2003 or at the time of their most recent follow-up prior to this date. Cumulative HIV incidence rates were calculated using the Kaplan-Meier methods and HIV-infection rates were compared by the log-rank test.

Cox proportional hazards regression was used to assess the independent effect of both fixed and time-dependent covariates on time to HIV seroconversion. In the first multivariate model, we considered all variables that were statistically associated with HIV seroconversion at the *p *< 0.05 level in univariate analyses in a fixed model that included all of these covariates. Because we were conscious that the number of HIV seroconversions was small and statistical power was limited, we also prepared a parsimonious model that only included those behavioral variables that remained statistically associated (*p *< 0.05) with HIV seroconversion after adjustment. All statistical analyses were performed using SAS software version 8.0 (SAS, Cary, NC). All reported *p *values were 2-sided.

## Results

Between May 1996 and May 2003, 1548 participants were recruited into the VIDUS study among whom there were 291 (19%) participants aged ≤24 years. Overall, 80 (27%) of the youth were Aboriginal and 211 (73%) were non-Aboriginal.

As shown in Table 1, there was no statistical difference between Aboriginal and non-Aboriginal youth with respect to age [median 16 (IQR: 14–18) vs. 17 (IQR: 15–19) respectively (*p *= 0.114)]; however, Aboriginal youth had, on average, been injecting longer [median number of years since first injection was 5 (IQR: 2–8) vs. 3 (IQR: 1–5)] than non-Aboriginal youth (*p *=< 0.001). Aboriginal youth were more likely to be female (OR: 2.22 [CI: 1.31–3.79]), report a history of sexual abuse (OR: 1.78 [CI: 1.06–2.99]), work in the sex trade (OR: 2.90 [CI: 1.70–4.94]), report a recent STI (OR: 1.73 [CI: 1.03–2.93]) and inject cocaine (OR: 2.00 [CI: 1.17–3.42]) and speedballs (OR: 2.07 [CI: 1.06–4.02]) on a frequent basis.

**Table 1:** Comparison of baseline sociodemographic characteristics, drug and sexual risk variables between Aboriginal youth (N = 80) and Non-Aboriginal youth (N = 211) aged 24 and under in the VIDUS project.

	Aboriginal Youth (N = 80, 27%)	Non-Aboriginal Youth (N = 211, 73%)	Odds Ratios [95% CI]	*p-value
**Years Fixing**	5 (IQR: 2–8)	3 (IQR: 1–5)	-------	<0.001
**HIV-positive**	16 (20)	20 (7)	4.15 [1.86–9.22]	<0.001
**HCV-positive**	53 (66)	80 (38)	3.21 [1.87–5.52]	<0.001
Female	52 (65)	96 (46)	2.22 [1.31–3.79]	0.003
**Unstable House**	52 (65)	124 (59)	1.30 [0.76–2.22]	0.332
**Sex Trade**	50 (63)	77 (36)	2.90 [1.70–4.94]	<0.001
**Sexual Abuse**	40 (50)	76 (36)	1.78 [1.06–2.99]	0.029
**Condoms w/Regular**	17 (21)	34 (16)	1.40 [0.73–2.69]	0.304
**Condoms w/Casual**	15 (19)	64 (30)	0.53 [0.28–1.00]	0.047
**STI**	37 (46)	70 (33)	1.73 [1.03–2.93]	0.039
**Alcohol Use**	34 (43)	88 (42)	1.03 [0.61–1.74]	0.903
**≥ 1 Daily Heroin**	38 (48)	119 (56)	0.70 [0.42–1.17]	0.174
**≥ 1 Daily Cocaine**	35 (44)	59 (28)	2.00 [1.17–3.42]	0.010
**≥ 1 Daily Crack**	16 (20)	26 (12)	1.78 [0.90–3.53]	0.096
**Help Injecting**	36 (45)	115 (55)	0.68 [0.41–1.15]	0.145
**On MMT**	0 (0)	7 (3)	-----------	0.099

*All reported p-values are two-sided. STI = sexually transmitted infection, MMT = methadone maintenance therapy.

There were no differences between Aboriginal and non-Aborginal groups with respect to alcohol use (OR: 1.03 [CI: 0.61–1.74]), condom use with regular sexual partners (OR: 1.40 [CI: 0.73–2.69]), crack cocaine use (OR: 1.78 [CI: 0.90–3.53]) or ever accessing methadone maintenance therapy (OR: 0.0 [CI: 0.0–0.0]). Aboriginal youth were less likely to use condoms with casual sexual partners (OR: 0.53 [CI: 0.28, 1.00]). Of note, at study enrollment, Aboriginal youth were more like to have tested HIV-positive (OR: 4.15 [95% CI: 1.86–9.22]) and HCV-positive (OR: 3.21 [CI: 1.87–5.52]).

In the multi-variable analysis that adjusted for all variables significant in the univariate analysis, independent associations with Aboriginal ethnicity were HIV positivity (OR: 2.54 [CI: 1.25–5.16]), HCV positivity (OR: 1.91 [CI: 1.00–3.65]), and sex trade involvement (OR: 2.08 [CI: 1.15–3.77]). Conversely, daily heroin injection was inversely associated with Aboriginal ethnicity (OR: 0.50 [CI: 0.28–0.91]). The multivariate model was further adjusted for residing in the Downtown Eastside of Vancouver (OR; 2.17 [CI: 1.21–3.88]) since this neighborhood is where injection drug use activity is concentrated and due to the disproportionate number of Aboriginal peoples residing in this area of Vancouver.

**Figure 1:**
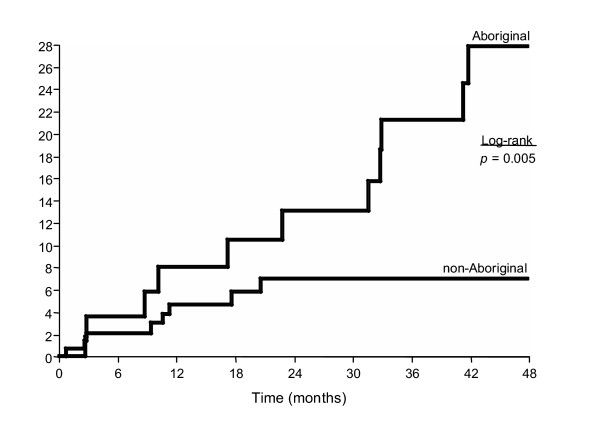
Kaplan-Meier product limit cumulative HIV incidence stratified by Aboriginal ethnicity.

We then examined the time to HIV-infection among Aboriginal and non-Aboriginal youth. There were 196 youth who were HIV negative at enrolment and completed at least one follow-up visit during the observation period. Of the 196 youth, 55 (28%) were Aboriginal and 141 (72%) non-Aboriginal. As of May 31, 2003, HIV seroconversion had occurred in 21 (11%) of the 196 youth, among whom 12 (22%) were among Aboriginal and 9 (6%) were among non-Aboriginal youth. As shown in the Kaplan-Meier analysis (Figure 1) the cumulative HIV incidence rate after 48 months was 27.8% (95% CI: 13.4–42.2) for Aboriginal youth vs. 7.0% (CI: 2.3–11.8) for non-Aboriginal youth (log-rank *p *= 0.005) and HIV incidence density over the entire follow-up period was 12.6% per 100 pyrs (CI: 6.49–21.96) and 3.9 per 100 pyrs (CI: 1.8–7.3) respectively.

In univariate Cox regression analyses, factors associated with HIV seroconversion among the youth were; Aboriginal ethnicity (Relative Hazard [RH]: 3.38 [95%CI: 1.43–8.03]), unstable housing (RH: 2.59 [CI: 1.00–6.71]), sex trade (RH: 2.96 [CI: 1.24–7.07]), ≥1 daily heroin injection (RH: 2.65 [CI: 1.05–6.73]), ≥1 daily cocaine injection (RH: 5.85 [CI: 2.40–14.30]), and requiring help injecting (RH: 3.47 [CI: 1.46–8.29]).

In the multi-variable analysis (Table 2), after removing non-significant (*p *> 0.05) behavioral variables, factors independently associated with HIV seroconversion among the youth were Aboriginal ethnicity (Adjusted Relative Hazard [ARH]: 2.46, [CI: 1.00–6.03]) and ≥1 daily cocaine injection (ARH: 3.88, [CI: 1.54–9.78]).

**Table 2:** Cox regression of the prognostic factors associated with time to HIV infection among the youth (aged ≤24) in the VIDUS cohort (N = 196).

**Characteristic**	**Relative Hazard [95% CI]**	**Adjusted Relative Hazard [95% CI]**
**Aboriginal**	3.38 [1.43–8.03]	2.46 [1.00–6.03]
**Female**	1.95 [0.79–4.84]	
**Unstable House**	2.59 [1.00–6.71]	2.20 [0.84–5.76]
**Sex Trade**	2.96 [1.24–7.07]	
**Condoms w/Reg**	0.20 [0.03–1.52]	
**Condoms w/Casual**	0.83 [0.30–2.28]	
**≥ 1 Daily Heroin**	2.65 [1.05–6.73]	
**≥ 1 Daily Cocaine**	5.85 [2.40–14.30]	3.88 [1.54–9.78]
**Help Injecting**	3.47 [1.46–8.29]	2.18 [0.89–5.76]
**On MMT**	1.66 [0.64–4.30]	

## Discussion

The main finding of this study was that Aboriginal youth who inject drugs were more than four times as likely to be HIV-infected at enrolment and were more than twice as likely to become HIV-infected during follow-up than non-Aboriginal youth who inject drugs. These data are concerning and corroborate the concerns voiced by many people in the Aboriginal community as well as Aboriginal service providers[8]. The exceedingly high HIV incidence rate of 27.8 per person years among the young Aboriginal participants indicates that culturally appropriate prevention programs and services that address the health needs of HIV-positive Aboriginal people are urgently needed.

Aboriginal youth were more likely to inject cocaine on a daily basis than non-Aboriginal youth. Injection cocaine use has consistently been found to be a strong independent risk factor for HIV and HCV infection among IDUs[16], particularly in this setting[8,11,17]. Aboriginal youth were more likely to use crack on a daily basis, a behaviour that has been shown to increase vulnerability to sexually transmitted infections [18-20].

None of the young Aboriginal injectors had ever accessed methadone maintenance therapy even though almost half reported using heroin daily at baseline. Methadone maintenance therapy has been shown to aid in risk reduction among injection drug users[21,22]. Overall, uptake of methadone among the young participants is very low suggesting there may be a need to explore reduced access to methadone treatment services among at risk youth. In the United States some studies have suggested that African-American IDU are less likely to be enrolled in methadone maintenance programs [23-25] which may be due in part to African American peoples distrust of methadone as a substance abuse treatment[26]. This finding may also suggest an apprehension among Aboriginal youth to access treatment services not specifically designed for and in collaboration with this population.

Among the Aboriginal youth in this study, 65% were female, which is disproportionate to other IDU cohort studies where females tend to comprise approximately one third of the sample[13,16,27]. The link between young Aboriginal females and injection drug use in the present setting requires further investigation; at the very least, this finding underscores a need for targeted interventions designed specifically for and in collaboration with young Aboriginal females. In this study, Aboriginal youth were more likely to be engaged in sex trade work and to report a history of sexual abuse, which may be explained by the higher proportions of females in this group. However, there remains an urgent need for a culturally appropriate public health response to sexual abuse survivors and sex work prevention programs for children as well as programs offering safety to young women involved in sex work.

Compared to other youth, Aboriginal youth were more likely to report lower prevalence of condom use with casual sexual partners and to self-report recently diagnosed STI's. These findings demonstrate a potential need for increased awareness regarding the risks of HIV acquisition from unsafe sex in this population. HIV interventions among this population will likely need to consider differential power dynamics between females and males in sexual relationships as well as the effects of discrimination, the residential school system and cultural dislocation [28-30].

Several limitations of this study should be acknowledged. First, as has been previously described, the study population was not a random sample of all IDUs and the analyses was primarily based on self-reported behaviours. However, previous studies in our setting have suggested the sample is representative of IDUs in the community[31]. Nevertheless, due to the small sample size in our analyses, further studies will be required to confirm the risk factors associated with HIV seroconversion among Aboriginal youth. In addition, qualitative studies will likely be valuable in providing a better understanding of the HIV-related vulnerabilities that may be unique to young Aboriginal people.

In summary, we identified exceedingly high baseline HIV prevalence and subsequent elevated HIV incidence among young Aboriginal injection drug users. Our study also highlights the disproportionate number of young female Aboriginal people using injection drugs and an increased vulnerability to sex work. Our findings demonstrate the urgent need for policy-makers, in collaboration with the affected community, to implement an evidence-based and culturally appropriate HIV prevention and addiction treatment strategy to respond to the dual epidemics of injection drug use and HIV among young Aboriginal drug users. Ultimately, all service delivery programs including drug treatment and methadone maintenance therapy, requires Aboriginal involvement to validate and provide culturally appropriate assessments of the services offered.
